# Do internationally adopted children in the Netherlands use more medication than their non-adopted peers?

**DOI:** 10.1007/s00431-016-2697-7

**Published:** 2016-02-05

**Authors:** Joost R. van Ginkel, Femmie Juffer, Marian J. Bakermans-Kranenburg, Marinus H. van IJzendoorn

**Affiliations:** Centre for Child and Family Studies, Leiden University, P.O. Box 9555, 2300 RB Leiden, The Netherlands

**Keywords:** Antidepressants, Medication for ADHD, Contraception, Medication for growth stimulation, Medication for precocious puberty

## Abstract

Empirical evidence has shown that international adoptees present physical growth delays, precocious puberty, behavioral problems, and mental health referrals more often than non-adoptees. We hypothesized that the higher prevalence of (mental) health problems in adoptees is accompanied by elevated consumption of prescription drugs, including antidepressants, attention deficit hyperactivity disorder (ADHD) medication, and medication for growth inhibition/stimulation. In an archival, population-based Dutch cohort study, data on medication use were available from the Health Care Insurance Board by Statistics Netherlands from 2006 to 2011. The Dutch population born between 1994 and 2005 and alive during the period of measurement was included (2,360,450 including 10,602 international adoptees, of which 4447 from China). Their mean age was 6.5 years at start (range 1–12 years) and 11.5 years at the end of the measurement period (range 6–17 years). Chinese female adoptees used less medication for precocious puberty (as treatment for precocious puberty; odds ratio (OR) = 0.57, effect size Cohen’s *d* = −0.31) and contraception (OR = 0.65, *d* = −0.24) than non-adoptees. For both males and females, non-Chinese adoptees used more medication for ADHD than non-adoptees (males: OR = 1.22, females: OR = 1.32), but the effect was small (males: *d* = 0.11, females: *d* = 0.15).

*Conclusions*: Adoptees in the Netherlands generally do not use more medication than their non-adopted peers.

**Table:** 

**What is Known:** • *Meta-analytical evidence shows that international adoptees present physical growth delays and mental health referrals more often than non-adopted controls.* • *With the exception of one Swedish study on ADHD medication, there is no other systematic research on medication use of international adoptees.*
**What is New:** • *All differences in medication use between international adoptees in the Netherlands and non-adopted controls were below the threshold of a small effect with the exception of medication for precocious puberty, but this effect was in the opposite direction with female adoptees using less medication for precocious puberty than non-adoptees.* • *International adoptees in the Netherlands do not use more medication despite experiences of preadoption adversity and higher rates of mental health referrals during childhood and adolescence.*

## Introduction

Internationally adopted children often face the consequences of inadequate prenatal and perinatal medical care, separations, psychological deprivation, insufficient health services, neglect, abuse, and malnutrition in orphanages or poor families before adoptive placement [8, 9, 17, 19–21, 29, 32]. A systematic meta-analysis [14] indeed found more mental health referrals (Cohen’s [2,3] *d* = 0.72), more externalizing (*d* = 0.24), and internalizing (*d* = 0.16) behavioral problems in adoptees than in non-adoptees, although the latter two effects were small. McLaughlin et al. [18] showed that children reared in institutions—often a prestage of adoption—showed more symptoms of attention deficit hyperactivity disorder (ADHD) than controls growing up in their biological families (see also Stevens et al. [25]). In a study on adopted children in five countries, Roskam et al. [22] found that a longer exposure to deprivation in institutions coincided with an increase in ADHD symptoms. Adopted children may also present physical health problems [19]. The more time children spend in institutional care, the more they lag behind in height, weight, and head circumference [28]. In a Danish study, adopted girls had a 10 to 20 times higher risk of precocious puberty than non-adopted girls [26].

The higher incidence of mental and physical health problems in adoptees may result in elevated use of medication. To our knowledge, no research on this topic has been conducted yet, except by Lindblad, Weitoft, and Hjern [16], who found that in Sweden, ADHD medication was more frequent among international adoptees than among non-adoptees. In this study, only ADHD medication was examined. In the current study, we include a broader range of medical prescriptions: antidepressants (as a remedy for internalizing problems), ADHD medication, contraception (more contraception may be used by adoptees because of precocious puberty and possible behavior problems), medication stimulating growth (as a remedy for growth delay), and medication inhibiting growth (as a remedy for precocious puberty). The central question is: Do adoptees and non-adoptees in the Netherlands differ in medication use? We make a distinction between adoptees from China and adoptees from other countries, because in the Netherlands, the majority of adoptees came (and still come) from China, and worldwide China has been the country sending most children (predominantly girls) for international adoption since 1995 [24]. Adoption from China was largely rooted in the one-child policy during the years studied in the current investigation. Consequently, Chinese adoptees usually did not experience prenatal adversities, in contrast with adoptees from other countries where parental drug or alcohol dependence or extreme poverty might lead to relinquishment or abandonment. Still, Chinese adoptees experienced separation from their biological parents and potential preadoption adversity, and we therefore expect to find more medication use in a nation-wide cohort of adoptees compared to their same-aged peers during a 6-year registration period.

## Method

### Data

Data on medication use (prescribed medicines dispensed by pharmacies) were available from 2006 to 2011 (measurement period) for the complete Dutch population. They were collected from the Health Care Insurance Board (College voor Zorgverzekeringen) by Statistics Netherlands (Centraal Bureau voor de Statistiek). The information on medication usage came from the Healthcare Insurance Board (College voor Zorgverzekeringen, CVZ) and consisted of information from the Dutch national registry of pharmacies, which is independent of the setting or location where the children would receive the medication. All prescriptions are automatically included in the database; no information was given by respondents themselves. Background variables such as age and gender came from non-public microdata from Statistics Netherlands and were largely collected from external bureaus but also partly by primary observation using surveys. Occasionally, missing information has been imputed by Statistics Netherlands. Variables concerning adoption were obtained by Statistics Netherlands using information from the Dutch Immigration and Naturalisation Service (Immigratie-en Naturalisatiedienst) and from the Municipal Personal Records Database (Gemeentelijke Basisadministratie, GBA). Finally, information on income was collected by Statistics Netherlands and came from several administrations. The most important provider is the tax collectors office (Belastingdienst).

All members of the Dutch population, who were born between 1994 and 2005 and were alive during the complete period of measurement, were included. This cohort includes all children with ages between birth and late puberty during the period of measurement (2006–2011). The youngest children were younger than 1 year of age at the start while the oldest children were 17 years old at the end. The population of interest was *N* = 2,360,450, including *n* = 10,602 international adoptees and excluding *n* = 384 domestic adoptees.

### Predictor variables

The predictor variables were the usage of five medication categories. These five categories and the medicines that fall into these categories are listed in Table 1. Classification was done by a medical expert. For each type of medicine, usage was measured as the number of years the specific medicine category was used during the measurement period.

**Table 1:** Overview of all medication categories and their contents

Medication category	Medicines
Medication for ADHD	Psychostimulants
	Agents used for ADHD
	Nootropics
Contraception	Contraceptives
	Sex hormones and modulators of the genital system
	Estrogens with progestogens
Medication for growth stimulation	Androgens
	Anterior pituitary lobe hormones
	Hypothalamic hormones
Growth inhibitors as treatment for precocious puberty	Estrogens
Other	All other medicines.

### Background variables

Background variables were gender, age at the start of the measurement period, and gross household income.

### Dependent variable

The dependent variable was international adoption, classified in two categories (adopted and non-adopted).

### Statistical analyses

Two series of logistic regression analyses were carried out. In the first, adoption from countries other than China versus non-adoption was the outcome variable (excluding adoptees from China). In the second series of analysis, adoptees from China were compared with non-adoptees (excluding adoptees from other countries). The predictors in the analyses were all medication categories except contraception (which is only used by women), gender, year of birth, and all two-way interactions between gender on the one hand and the medication categories on the other hand. Data inspection revealed that growth inhibitors were hardly used by males, so the interaction Gender × Growth inhibitors was excluded from this analysis. In the analyses including interactions, predictors were centered. Additional analyses were carried out with the same predictors and outcome variables but for males and females separately; for females, contraception was included as an additional predictor.

Chinese adoptees were on average younger than non-adoptees and adoptees from other countries. To examine the influence of age on the outcomes, all analyses were carried out with and without controlling for age at start of the measurement period. Finally, we checked whether age at adoption (as a proxy variable for duration of adversity) [19, 21, 29] influenced the outcomes.

It should be noted that substantively, it would make sense to consider adoption as a predictor variable rather than as an outcome variable, because we interpret adoption as the “causal” factor that might elevate use of medication. To enable multivariate analyses, we decided to use adoption as an outcome. Note that the statistical techniques examine associations between variables and remain agnostic about their role in a causal model. Other analyses, such as multilevel logistic regression with medication use (use/non-use) as an outcome variable, and medication categories and time points nested within individuals were not used because of computational problems and poor performance of estimation methods for multilevel logistic regression models in general (e.g., [1]).

## Results

### Descriptive statistics

The mean age of the population was 6.5 years at the start (range 1–12 years) and 11.5 years at the end of the measurement period (range 6–17 years). Table 2 shows the mean age for non-adoptees, adoptees from China, and adoptees from other countries separately, along with descriptive statistics of the other relevant variables. Most international adoptees came from China (*n* = 4,447, 42 %), followed by Colombia (*n* = 1,469, 14 %), Ethiopia (*n* = 556, 5 %), Taiwan (*n* = 514, 5 %), Haiti (*n* = 499, 5 %), India (*n* = 471, 4 %), South Korea (340, 3 %), Brazil (*n* = 309, 3 %), and other countries (*n* = 1,997, 19 %).

### Logistic regression analyses

Because in general, the regression coefficients with and without age at start and gross household income taken into account were highly similar, we present the results of the analyses that included age at start and gross household income as covariates. When regression coefficients are divergent, the results with and without both covariates are presented.

#### Adoptees not from China

The results are shown in Table 3. For the analyses including age at start and gross household income (Model 2), none of the logistic regression results met the criteria for a “small effect.” Since growth inhibitors were hardly used by males, they were only included in the analyses on females (Table 3). In the analyses for males and females separately, none of the effects met the criteria for a small effect (Table 4). The effect of medication for ADHD was significant (for the total group of adoptees and for males and females separately), but did not meet Cohen’s criteria for a small effect.

**Table 2:** Descriptive statistics for the predictor and background variables for the different subpopulations and for males and females separately

Group	Variable	*Male*		*Female*	
		*M*	*SD*	*M*	*SD*
Non-adopted		(*n =* 1,203,187)		(*n =* 1,146,661)	
	Age in years at start (2006)	6.501	3.424	6.504	3.425
	Age in years at end (2011)	11.501	3.424	11.505	3.425
	Gross household income (2006)^a^	6.661	4.697	6.660	4.689
	Usage antidepressant in years	0.013	0.183	0.012	0.166
	Usage medication ADHD in years	0.246	0.971	0.069	0.488
	Usage contraception in years	–	–	0.228	0.694
	Usage growth stimulators in years	0.012	0.197	0.009	0.180
	Usage growth inhibitors in years	0.000	0.014	0.005	0.097
	Usage other medication in years^b^	2.811	1.894	2.890	1.841
Chinese adoptees		(*n =* 457)		(*n =* 3990)	
	Age in years at start (2006)	5.123	2.645	5.390	2.749
	Age in years at end (2011)	10.123	2.645	10.390	2.749
	Gross household income (2006)^a^	8.018	5.014	8.501	5.150
	Usage antidepressant in years	0.026	0.218	0.005	0.084
	Usage medication ADHD in years	0.256	0.933	0.042	0.338
	Usage contraception in years	–	–	0.064	0.337
	Usage growth stimulators in years	0.018	0.186	0.013	0.221
	Usage growth inhibitors in years	–	–	0.003	0.052
	Usage other medication in years^b^	3.315	1.938	3.022	1.901
Other adoptees		(*n* = 3387)		(*n =* 2768)	
	Age in years at start (2006)	7.051	3.073	7.207	2.995
	Age in years at end (2011)	12.051	3.073	12.207	2.995
	Gross household income (2006)^a^	7.751	4.954	7.848	5.368
	Usage antidepressant in years	0.027	0.258	0.032	0.304
	Usage medication ADHD in years	0.593	1.488	0.227	0.911
	Usage contraception in years	–	–	0.281	0.786
	Usage growth stimulators in years	0.021	0.282	0.024	0.306
	Usage growth inhibitors in years	–	–	0.004	0.060
	Usage other medication in years^b^	3.028	1.986	3.033	1.921

^a^Measured in 10,000 Euros per year

^b^The average usage of this miscellaneous category is substantially larger than the other categories because it includes all other medication

**Table 3:** Results of the logistic regression analysis with adoption not from China versus non-Adoption as the dependent variable, males and females together

Effect	Model 1				Model 2			
	*b*	SE	OR	Effect size (*d*)	*b*	SE	OR	Effect size (*d*)
Intercept	−6.021	0.019***	0.002		−6.056	0.019***	0.002	
Gender (ref. cat. = female)	0.094	0.026***	1.098	0.052	0.098	0.026***	1.103	0.054
Antidepressants	0.121	0.087	1.128	0.067	0.090	0.085	1.094	0.050
Medication ADHD	0.296	0.021***	1.344	0.163	0.282	0.020***	1.326	0.155
Growth stimulators	0.018	0.090	1.018	0.010	0.041	0.086	1.042	0.023
Growth inhibitors	−0.329	0.260	0.720	−0.181	−0.339	0.257	0.712	−0.187
Other	0.033	0.010**	1.034	0.018	0.041	0.010**	1.042	0.023
Antidepressants × gender	−0.059	0.116	0.943	−0.033	−0.041	0.113	0.960	−0.023
Medication ADHD × gender	−0.081	0.023***	0.923	−0.045	−0.080	0.023***	0.923	−0.044
Growth stimulators × gender	−0.017	0.120	0.983	0.009	−0.041	0.115	0.960	0.023
Other × gender	0.017	0.014	1.017	0.009	0.026	0.014	1.026	0.014
Age at start (2006)					0.046	0.004	1.047	0.025
Household income (2006)					0.038	0.002	1.039	0.021
	Nagelkerke’s *R* ^2^ = 0.007				Nagelkerke’s *R* ^2^ = 0.007			

*p < 0.05; **p < 0.01; ***p < 0.001

#### Adoptees from China

In the analysis with Chinese adoptees (Table 5, Model 2), males had a lower probability of having been adopted than females (OR = 0.11). This means that adopted children from China are more often female than male, which is not surprising considering China’s one-child policy [11]. Growth inhibitors were used less frequently by Chinese adoptees than non-adoptees (OR = 0.58). Moreover, antidepressants were used less frequently by the Chinese adoptees than their non-adopted peers (OR = 0.67), but this effect was qualified by a significant interaction with gender. Females adopted from China seemed to use fewer antidepressants than their non-adopted peers, whereas males adopted from China seemed to use more antidepressants than their non-adopted peers (see Table 5). In analyses for males and females separately, however, no substantial effects of antidepressants emerged (Table 6, Model 2). In the analyses for males and females separately, females used fewer growth inhibitors than their non-adopted peers (OR = 0.61). In the analysis for females, the effect of contraception was significant both with (Model 2) and without (Model 1) age at start and gross household income taken into account. In both models, females adopted from China used less contraception than non-adopted females of their age. However, this effect was substantially larger when age at start and gross income were not included (OR = 0.49) than when age at start and gross income were included (OR = 0.62; 85 % confidence intervals not overlapping). For males, none of the effects were of discernible size (Table 6).

**Table 4:** Results of the logistic regression analyses with adoption not from China versus non-adoption as the dependent variable as the dependent variable, for males and females separately

Gender	Effect	Model 1				Model 2			
		*b*	SE	OR	Effect size (*d*)	*b*	SE	OR	Effect size (*d*)
Male	Intercept	−6.106	0.033***	0.002		−6.683	0.055***	0.001	
	Antidepressants	0.062	0.077	1.064	0.034	0.052	0.075	1.053	0.029
	Medication ADHD	0.215	0.011***	1.240	0.119	0.204	0.011***	1.226	0.112
	Growth stimulators	0.001	0.079	1.001	0.001	−0.001	0.077	0.999	−0.001
	Other	0.050	0.009***	1.052	0.028	0.066	0.009***	1.068	0.036
	Age at start (2006)					0.039	0.005	1.040	−0.022
	Gross household income (2006)					0.038	0.003	1.039	0.021
		Nagelkerke’s *R* ^2^ = 0.007				Nagelkerke’s *R* ^2^ = 0.012			
Female	Intercept	−6.168	0.037***	0.002		−6.875	0.061***	0.001	
	Antidepressants	0.097	0.090	1.102	0.053	0.120	0.082	1.128	0.066
	Medication ADHD	0.294	0.021***	1.341	0.162	0.281	0.020***	1.324	0.155
	Growth stimulators	0.005	0.093	1.005	0.003	0.066	0.086	1.068	0.036
	Growth inhibitors	−0.305	0.258	0.737	−0.168	−0.320	0.254	0.726	−0.176
	Contraception	0.046	0.026	1.048	−0.025	−0.078	0.029	0.925	−0.043
	Other	0.031	0.010**	1.031	0.017	0.046	0.010**	1.047	0.025
	Age at start (2006)					0.017	0.061**	1.063	0.009
	Gross household income (2006)					0.038	0.003	1.039	0.021
		Nagelkerke’s *R* ^2^ = 0.005				Nagelkerke’s *R* ^2^ = 0.011			

*p < 0.05; **p < 0.01; ***p < 0.001

**Table 5:** Results of the logistic regression analysis with adoption from China versus non-adoption as the dependent variable, males and female together

Effect	Model 1				Model 2			
	*b*	SE	OR	Effect size (*d*)	*b*	SE	OR	Effect size (*d*)
Intercept	−5.682	0.017	0.003		−5.774	0.018	0.003	
Gender (ref. cat. = Female)	−2.224	0.051***	0.108	−1.226	−2.234	0.051**	0.107	−1.232
Antidepressants	**−0.525**	0.183**	**0.592**	**−0.289**	**−0.404**	0.176*	**0.668**	**−0.223**
Medication ADHD	−0.141	0.044**	0.869	−0.078	−0.096	0.045	0.909	−0.053
Growth stimulators	0.203	0.067	1.225	0.112	0.211	0.069	1.235	0.116
Growth inhibitors	**−0.502**	0.255*	**0.605**	**−0.277**	**−0.540**	0.259*	**0.583**	**−0.298**
Other	0.042	0.009	1.042	0.023	0.046	0.009	1.047	0.025
Antidepressants × gender	**0.713**	0.269**	**2.041**	**0.393**	**0.661**	0.273*	**1.936**	**0.364**
Medication ADHD × gender	0.130	0.066	1.139	0.072	0.138	0.067	1.148	0.076
Growth stimulators × gender	−0.240	0.236	0.786	0.132	−0.303	0.253	0.739	0.167
Other × gender	0.096	0.026	1.101	0.053	0.079	0.026	1.082	0.044
Age at start (2006)					−0.100	0.005	0.905	−0.055
Gross household income (2006)					0.054	0.002	1.055	0.030
	Nagelkerke’s *R* ^2^ = 0.070				Nagelkerke’s *R* ^2^ = 0.070			

Effects with |*d*| ≥ 0.20 are printed in bold

*p < 0.05; **p < 0.01; ***p < 0.001

#### Controlling for age at adoption within group of adoptees

In earlier research [28, 29], it was found that length of early deprivation, indicated by age at adoption, is a predictor for delays in physical growth and school achievement in adoptees. To see to what extent differences in results between adoptees from China and adoptees from other countries could have been attributed to possible differences in age at adoption, logistic regression analyses were performed on the total group of adopted children with all predictors of the other analyses, but with age at adoption as a mediator variable (not tabulated). Here, adoption from China versus adoption from other countries was the outcome variable (ref. category = China). In the analysis with boys and girls together, no significant main effect of age at adoption was found. In the analysis with boys only, it was found that as age at adoption increased, adoptees were more likely to be adopted from China than from other countries (*p* < 0.001, OR = 1.26), whereas for girls, the reverse was found (*p* < 0.001, OR = 0.92). However, adding age at adoption as a mediator variable did not alter the effect sizes of the medication effects in any way, compared to the same analyses without age at adoption as a mediator variable.

#### Analyses with adolescents only

Some medications such as contraceptives or antidepressants may not be given to young children. This raises the question whether the results of the above analyses would have been different if only adolescents were included. To test this, all of the above analyses were carried out again, using only children who were born between 1994 and 1996 and aged 10 to 17 years in the measurement period (not tabulated).

These analyses showed only a few changes in the results. Firstly, for the analysis with adopted females not from China versus non-adopted females (Table 4, lower panel), the effect of antidepressants became significant in Model 2 when only adolescents were included (*b* = 0.19, *p* = 0.04, OR = 1.21, *d* = 0.11). However, this effect did not meet the requirements for a small effect (Cohen’s |*d*| ≥ 0.20).

**Table 6:** Results of the logistic regression analyses with adoption from China versus non-adoption as the dependent variable, for males and females separately

Gender	Effect	Model 1				Model 2			
		*b*	SE	OR	Effect size (*d*)	*b*	SE	OR	Effect size (*d*)
Male	Intercept	−8.297	0.094***	0.000		−7.917	0.140***	0.000	
	Antidepressants	0.188	0.198	1.207	0.104	0.266	0.211	1.305	0.147
	Medication ADHD	−0.011	0.049	0.990	−0.006	0.050	0.050	1.051	0.028
	Growth stimulators	−0.037	0.226	0.963	−0.020	−0.098	0.248	0.906	−0.054
	Other	0.138	0.025***	1.147	0.076	0.118	0.025***	1.125	0.065
	Age at start (2006)					−0.117	0.015***	0.890	−0.065
	Gross household income (2006)					0.047	0.007***	1.048	0.026
		Nagelkerke’s *R* ^2^ = 0.004				Nagelkerke’s *R* ^2^ = 0.017			
Female	Intercept	−5.736	0.030***	0.003		−5.724	0.046***	0.003	
	Antidepressants	−0.163	0.172	0.849	−0.090	−0.144	0.172	0.866	−0.079
	Medication ADHD	−0.119	0.044**	0.888	−0.066	−0.090	0.044	0.914	−0.050
	Growth stimulators	0.211	0.065**	1.235	0.116	0.216	0.067	1.241	0.119
	Growth inhibitors	**−0.503**	0.256*	**0.605**	**−0.277**	**−0.533**	0.258*	**0.587**	**−0.294**
	Contraception	**−0.706**	0.050***	**0.494**	**−0.389**	**−0.483**	0.051***	**0.617**	**−0.266**
	Other	0.058	0.009***	1.060	0.032	0.057	0.009***	1.059	0.031
	Age at start (2006)					−0.074	0.005***	0.928	−0.041
	Gross household income (2006)					0.054	0.002	1.055	0.030
		Nagelkerke’s *R* ^2^ = 0.008				Nagelkerke’s *R* ^2^ = 0.020			

Effects with |*d*| ≥ 0.20 are printed in bold

*p < 0.05; **p < 0.01; ***p < 0.001

Secondly, for the analysis with adoptees from China versus non-adoptees (Table 5), the interaction of other medication and gender was significant in both models (Model 1: *b =* 0.25, *p* = 0.01, OR = 1.29, *d* = 0.14; Model 2: *b* = 0.25, *p* = 0.01, OR = 1.28, *d* = 0.14). Again, this effect had no discernible size. Also, in this subpopulation, there were so few males using growth simulators that the interaction of gender and growth stimulators was left out of this analysis, and the main effect of growth stimulators was left out for the analysis with males only.

Thirdly, in the analyses with adopted children from China versus adopted children not from China with age at adoption as a moderator variable, the results changed little when only adolescents were included. In the analysis with boys and girls together, the odds ratio for the main effect of age at adoption changed from OR = 1.00 (*p* = 0.91) to OR = 0.83 (*p* < 0.001) but the effect was not of discernible size. In the same analysis, the interaction of other medications and gender became significant, going from OR = 1.05 (*p* = 0.09; complete sample) to 1.29 (*p* = 0.01, adolescents only). However, this effect did not meet the requirements for a discernible effect size.

Finally, for the analysis with females only, the effect of other medications became significant, going from OR = 1.00 (*p* = 0.705; complete sample) to OR = 0.93 (p < 0.05; adolescents only). This effect did not meet the requirements for a discernible effect size, either.

#### Identifying subgroups of heavy medication users among adoptees

The work of Hjern, Lindblad, and Vinnerljung [10] has shown that most adoptees do well in terms of serious behavior problems, but that there is a subset of individuals with serious problems. This raises the question whether, in our population, there could be a subgroup of heavy users of medication as well. To answer this question, it was investigated for each time point (years 2006–2011) whether the number of medications used differed across the different genders, different birth years (1994, 1995, 1996), different adoption groups (non-adoptees, adoptees from China, adoptees not from China), or combinations of different levels of these factors. To this end, a 6 (Time point) × 2 (Gender) × 3 (Birth year) × 3 (Adoption group) ANOVA was carried out with the number of medications used (except for contraceptives which is used by females only; possible range of number of medications used 0 to 4) as the outcome variable. Time point was a within-subjects factor; the other factors were between-subjects factors. In this ANOVA, especially the interaction effects of the Adoption group with the other factors were relevant for identifying subgroups of heavy users.

The ANOVA revealed two significant interactions of the Adoption group with other factors, namely of Adoption group × Gender, *F*(2, 580846) = 6.66, *p* < 0.01, partial *η*^2^ = 0.00), and of Adoption group × Gender × Birth year, *F*(4, 580846) = 3.51, *p* = 0.01, partial *η*^2^ = 0.00). Interpreting the former interaction, non-adopted males (*M* = 0.50, *SE* = 0.00) used on average less medication than non-adopted females (*M* = 0.53, *SE* = 0.00), males adopted from countries other than China (*M* = 0.60, *SE* = 0.01) used about as much medication as females adopted from countries other than China (*M* = 0.60, *SE* = 0.01), but males adopted from China (*M* = 0.69, *SE* = 0.07) used on average *more* medication than females adopted from China (*M* = 0.49, *SE* = 0.01). The three-way interaction effect will not be further discussed because of the complexity of three-way interactions in general, but it should be noted that this interaction showed that boys adopted from China stood out with respect to medication use compared to the other groups as well. However, neither of these effects was sizeable.

## Discussion

In a large population-based cohort study (*N* = 2,360,450 children, mean age 6.5 years at the start and 11.5 years at the end of the measurement period; range 1 to 17 years), we examined medication use in international adoptees from China (*n* = 4,447) and from other countries of origin (*n* = 6,155), and compared them to non-adopted peers. We found that females adopted from China used fewer medication for precocious puberty than non-adopted females. Adoptees from other countries than China did not use substantially more (or less) medication than their non-adopted peers. Non-Chinese adopted children took somewhat more medication for ADHD than their non-adopted peers, but the effects (males and females together: *d* = 0.16; males: *d* = 0.12; females: *d* = 0.16) were below the threshold of a small effect (Cohen’s [2,3] *d* = 0.20). We found no sizeable differences between non-Chinese adoptees and non-adopted peers in their use of antidepressants, contraception (females), growth stimulators, or other medication. Carrying out the analyses for adolescents only did not change the results substantially.

In contrast to expectations, international adoptees do not use substantially more medication than their non-adopted peers. All differences in medication use between adoptees and non-adopted controls were below the threshold of a small effect, with the exception of medication for precocious puberty for adoptees from China, but this effect was in the opposite direction with female adoptees using less medication for precocious puberty. Our outcomes indicate that adoptees are not at risk for higher medication use despite possible adversity in their early childhood. The current outcomes converge with the absent or small delays that we found in a series of meta-analyses, pointing to a remarkable catch-up in all domains of adoptees’ physical and social-emotional development [14, 28, 29]. It should be noted that a small group of male adoptees from China use on average relatively many medications. However, this makes sense because in contrast to female Chinese adoptees, male adoptees from China often had special needs [34], which may require additional use of medication.

Previously, we found a large effect for mental health referrals and for educational and school support [14, 30], indicating that compared to biological families, adoptive parents seek more help and support for their adopted children [15]. The current outcomes suggest that adoptive parents actively seek psychological and educational support, but not medical treatment. The finding of more mental health referrals may also indicate that problems were indeed prevented or solved without (further) need of medication use. Adoptive parents may seek and find alternative ways to deal with the possibly difficult behavior of their adopted children. They were officially screened and prepared for their parenting job during the process of becoming adoptive parents. Due to this selection effect and the usually high socioeconomic status of adoptive parents, the threshold to seek psychological (rather than medical) treatment may be lower for adoptive parents than for biological families (see also Warren [33])^.^ However, this interpretation has been questioned by results from Swedish national population studies (e.g., [10]), and an alternative interpretation might relate to the potentially restricted mental health indicator of pharmacy-retrieved prescriptions of ADHD and antidepressant medications. These may provide a somewhat restricted picture of the prevalence of mental health problems among international adoptees that in some cases may include serious psychiatric disorders requiring in-patient treatment.

As an alternative explanation, medical practitioners may be reluctant to prescribe medication to internationally adopted children. This could be due to several factors, including the idea that adoptive parents will succeed in managing problems without resorting to medication or that the problems result from prior adversity and therefore may be unresponsive to medication. General practitioners may also assume that problem behaviors in adopted children will decrease with age, or that the children will catch up in development and thus will not require medical treatment. Unfortunately, it cannot be derived from our data which of the two explanations is most likely.

The use of fewer growth inhibitors in female adoptees (both from China and other countries) compared to non-adopted controls of the same age converges with the meta-analytical outcomes on the physical growth of international adoptees [28]. Adopted children show large delays in height, weight, and head circumference when they arrive, but after some years, the catch-up in height and weight is impressive, with head circumference showing somewhat less improvement. Although during childhood and adolescence adoptees usually catch up in height, they continue to lag behind their non-adopted peers and on average end with a shorter height [5]. Medication to delay the onset of puberty might be used in case adoptive parents are afraid of small stature, but we did not find substantially elevated use of growth-related medication. It should be noted however that precocious puberty is a rare medical condition. The adoptees or their parents seem to be satisfied with their substantial catch-up growth and to accept the remaining growth delays without turning to medical solutions.

Although adoptees have been found to show more ADHD than their non-adopted peers [22], in particular, the adoptees not from China (both males and females) only use slightly more medication for ADHD than non-adoptees. Our outcomes are in contrast with Lindblad et al. [16] who in a Swedish population-based cohort study found that the rates of ADHD usage were substantially higher in adoptees than in the reference population for both boys and girls of 10–15 years old. This may point at regional differences in medication use, varying diagnostic thresholds and diagnostic criteria, and differences in prevalence of behavior problems more generally (see for example Keyes et al. [15], who found moderately elevated levels of mental health issues in adoptees living in the USA). Regional differences may be caused by specific characteristics of the receiving country with diverging traditions of children’s medication use, or by different countries of origin of the adopted children, with diverging preadoption deprivation conditions.

Computing the effect sizes of Lindblad et al.’s [16] study resulted in large effect sizes for their total cohort (Cohen’s *d* = 0.83) (95 % confidence interval (CI) 0.55, 1.12), with similar effect sizes for boys (*d* = 0.71, CI 0.28, 1.14) and girls (*d* = 0.96, CI 0.53, 1.40). The substantial difference between ADHD medication use in Sweden versus the Netherlands might be related to differences in countries of origin of the adoptees. In Sweden, many come from deprived institutions in Eastern Europe and these children showed the highest risk for use of ADHD medication (see Lindblad et al. [16], p. 41), whereas in our study, the number of adoptees from Eastern Europe was very small (*n* = 474, 4 %; included in the category of “other countries”). The high risk for use of ADHD medication in Eastern European adoptees converges with the outcomes of several studies [6, 7, 9, 12, 17, 19–21, 23, 29] and a meta-analysis on attachment security [27] showing severe delays and difficulties in children adopted from Eastern Europe. In our study, the largest adoptee group was adopted from China as a consequence of the one-child-policy and probably without the experience of extreme adversity [31].

Based on the elevated rates of early puberty in adopted girls [26], increased rates of contraception use might be expected. However, in the current study, adopted girls did not use contraception more often than non-adopted girls, and the adopted girls from China even used less contraception (controlling for their somewhat younger age at the start of the measurement period). Although precocious puberty is related to early sexual maturation, it does not automatically translate into early sexual activity and relationships (implying the need for using contraception). Therefore, our finding may indicate that early puberty in adoptees is not a risk factor for early sexual activity and the need for contraception. In a similar vein, a Swedish register study showed that teenage childbirth was not more common among international female adoptees than among non-adopted controls [4]. It should also be noted that precocious puberty is quite rare, and associations with more widely used contraception are difficult to demonstrate.

Compared to the gender distribution of the non-adopted reference group, there were fewer adopted boys. This finding was mainly caused by the large number of female adoptees from China, which reflects adoption policy and practice in the recent past. In China, parents preferred a son over a daughter and abandoned female children more often than male offspring [11]. At the start of our measurement, the Chinese adoptees were on average younger than the other adoptees and the non-adopted peers, which can be explained by the fact that adoptions from China (of primarily very young children) started in the late 90s of the last century, whereas adoption from other countries started some decades earlier [24]. Recently, the population of adopted children worldwide has been changing, with more boys being adopted, and an increase in special-needs and older children [13].

Previous research [28, 29] showed that length of early deprivation, indicated by age at adoption, is a predictor for delays in physical growth and school achievement in adopted children. This raises the question to what extent differences in results between adoptees from China and adoptees from other countries could have been attributed to possible differences in age at adoption between both groups. However, in logistic regression analyses with adoption from China versus adoption from other countries as the outcome variable, none of the effects changed in size when age at adoption was added as a mediator variable. The same was the case when only adolescents were included in the analysis. Thus, differences in medication usage between adoptees from China and adoptees from other countries cannot be explained by differences in age at adoption.

Finally, a limitation of this study was that all information about the use of medication came from pharmacies. Using this source of information, some important treatments could have been missed, such as hormonal injections in a hospital setting, or other locations where the prescriptions were not entered into the database. It is unknown to what extent these missed cases of medication use could have influenced the results. However, it could be argued that in the Netherlands this influence may be relatively low because unlike in the USA, for example, the purchase of medication is rarely done through Internet because insurance companies will not reimburse medication that was purchased in other ways than through pharmacies.

We conclude that internationally adopted children in the Netherlands do not take substantially higher rates of antidepressants, ADHD medication, contraception (females), growth inhibitors/stimulators, or other medication than their non-adopted counterparts. Given the size of the current population-based cohort study, the power to find differences between adopted and non-adopted children was large. It seems therefore safe to conclude that adoptees do not use substantially more medication than their non-adopted peers. Our findings are in line with the remarkable catch-up growth shown by adopted children after arrival in a supportive family. Adoptees seem to recover from preadoption adversities without need for extra medical treatment.

## Abbreviations

ADHD: Attention deficit hyperactivity disorder
